# Reducing ageism among Israeli Jew and Arab middle school students: a randomized controlled trial

**DOI:** 10.1093/geront/gnaf274

**Published:** 2025-11-23

**Authors:** Assaf Suberry, Sarit Okun, Liat Ayalon

**Affiliations:** Louis and Gabi Weisfeld School of Social Work, Bar-Ilan University, Ramat Gan, Israel; Louis and Gabi Weisfeld School of Social Work, Bar-Ilan University, Ramat Gan, Israel; Louis and Gabi Weisfeld School of Social Work, Bar-Ilan University, Ramat Gan, Israel

**Keywords:** Ageism, Combating ageism, Educational intervention, RCT, Cross-cultural

## Abstract

**Background and Objectives:**

Ageism often emerges in childhood, yet rigorously evaluated school-based interventions—especially in multicultural settings—are scarce. The study evaluated the efficacy of a 90-min educational intervention to reduce ageism among Israeli Jewish and Arab middle school students.

**Research Design and Methods:**

Using a randomized controlled trial (RCT) design, 606 Israeli Jew and Arab middle school students (aged 12–16, 53.3% girls) were assigned to either an intervention (*N *= 314) or a control group (*N *= 292), with measures of stereotypes, prejudice, and discrimination collected at three time points.

**Results:**

In contrast to successful pilot findings, the intervention yielded no significant improvements over time on the measured outcomes. An exploratory analysis revealed that the pattern of change over time did not significantly differ across Israeli Jews and Arabs.

**Discussion and Implications:**

Results highlight challenges associated with reducing ageism in early adolescence. Our null findings contribute valuable knowledge that can guide future intervention design and advance cross-cultural ageism research.

## Introduction

Ageism encompasses the way individuals think (stereotypes), feel (prejudice), and behave (discrimination) based on chronological age. It can be directed toward other age groups or toward one’s own age group (WHO, 2021). Although ageism can affect people across the lifespan, it has been most extensively studied in relation to older persons, who are particularly vulnerable to its negative effects. Older persons are frequently subjected to negative treatment and are commonly viewed as weak, slow, and ill (Ayalon & Tesch-Römer, 2018). The COVID-19 pandemic has intensified ageism, with older persons often being portrayed in overly stereotypical terms—as frail, vulnerable, and as a burden (Apriceno et al., 2021; Cohn-Schwartz & Ayalon, 2021). In severe cases, older persons face discriminatory practices, financial exploitation, or abuse (Phelan & Ayalon, 2020; Weissberger, 2022).

Ageism can lead to internalized ageism, wherein individuals adopt and apply negative stereotypes about aging to themselves (Levy, 2009). These stereotypes are often deeply embedded within societal structures and are harmful across the lifespan, affecting both younger and older individuals. For children and younger adults, prevailing societal attitudes may foster intergenerational divides and contribute to distinct forms of age-based discrimination, including childism (prejudice against children), adultism (bias against adolescents and young adults), or eventually ageism in later life. In later life, negative portrayals of aging tend to become more elaborate, firmly entrenched, and readily accessible due to a lifetime of exposure to age-related biases. Simultaneously, age-related declines in cognitive control may reduce the ability to inhibit these internalized stereotypes (Henry et al., 2023).

Given its widespread impact, ageism poses both public health and economic challenges. Ageism has been shown to negatively affect older persons' physical health, well-being, and longevity (Hu et al., 2021; Kang & Kim, 2022; Levy et al., 2002). Ageism was also identified as a risk factor for chronic disease (Allen, 2016). Ageism also carries a significant financial burden, with estimates indicating that it contributes to approximately $63 billion in annual healthcare costs in the United States (Levy et al., 2020). As the global population of adults aged 65 and older continues to grow rapidly, addressing ageism has become an increasingly urgent social issue. For instance, recent meta-analytic evidence shows that ageism limits older persons' digital participation, reinforcing inequality and exclusion in later life (Huang & Chen, 2025). Other research has shown that analyses of 1,254 X posts during the 2024 U.S. campaign reveal pervasive ageist rhetoric—including dementia-related stigma that shifts attention from policies to age-based attacks, underscoring the societal reach of ageism (Bacsu et al., 2025).

Ageism starts early in life. Research indicates that children’s and tweens' perceptions of aging often reflect the predominantly negative societal views of older persons, although some positive beliefs may also be present (see Mendonça et al., 2018, for a review). A recent study found that children as young as 4–8 years consistently exhibited a preference for younger over older persons across explicit (picture rating), behavioral (seating and team formation task), and implicit measures (Jaquet et al., 2025). Understanding children’s views on aging is particularly important, as early stereotypes about older persons can develop into self-stereotypes over time, with documented negative consequences for individuals' mental and physical health in later life. Despite the significance of this issue, age-based attitudes in childhood and adolescence remain understudied relative to other domains such as race and gender.

Interventions to reduce ageism have been strongly advocated by the World Health Organization (WHO), which published a *Global Report on Ageism* in 2021. The WHO evidence-based report (2021) highlights three main strategies to reduce ageism: (a) policy and legislation; (b) intergenerational contact; and (c) educational interventions. The latter two have demonstrated the most promising results (Apriceno & Levy, 2023; Burnes et al., 2019). Notably, their efficacy is enhanced when they are combined, particularly when opportunities for meaningful contact between younger and older individuals are paired with structured education about aging and ageism.

## The prospect of educational interventions to reduce ageism

Theories of bias reduction provide valuable guidance for designing effective educational programs to reduce ageism. Social categorization theory (Tajfel & Turner, 1979) suggests that educational content should go beyond general messages of equality and instead actively restructure how youth perceive group boundaries—for example, by introducing counter-stereotypical portrayals of older persons that challenge assumptions of decline and dependence (Lai & Lisnek, 2023). At the same time, it is important to recognize that such changes are natural aspects of aging and that the stigma attached to them arises primarily from societal values rather than from the aging process itself. Devine et al.’s (2012) cognitive approach emphasizes the need for self-reflection activities that raise awareness of implicit age biases, helping students recognize and interrupt habitual stereotype use. In addition, Goldstein et al. (2008) highlights how peer norms shape individual attitudes. When peers express inclusive views, group discussions can create normative pressure that promotes stereotype reduction and more positive intergroup attitudes. Together, these frameworks underscore that ageism-reduction efforts must be multifaceted, addressing both cognitive and social dimensions of bias formation. Interventions designed in alignment with these principles may foster more enduring changes in youth attitudes toward older persons.

Indeed, education has been shown to play a significant role in reducing ageism. In a review of 58 studies, Chonody (2015) reported that 88% of the studies demonstrated positive shifts in attitudes following educational interventions. Educational initiatives targeting individuals across a wide range of age groups have been implemented globally, employing diverse strategies and formats. These interventions often focus on increasing knowledge about the aging process, thereby challenging myths and ageist stereotypes. Some interventions also raise awareness of how negative stereotypes about aging are socially embedded and frequently go unchallenged in public discourse. When participants are presented with factual, research-based information and are encouraged to engage in structured dialogue that explores conflicting attitudes, opportunities for critical self-reflection may arise (Devine et al., 2012). This process can foster meaningful changes in how individuals think, feel, and behave toward members of other age groups.

Educational interventions specifically targeting youth have demonstrated mixed efficacy depending on the mechanisms employed. Lichtenstein et al. (2001) found that integrating aging-related content into standard curricula (i.e., 12 sessions over 36 weeks) through practical exercises and real-world examples successfully reduced stereotypical attitudes toward aging among middle school students. Mellor et al. (2015) reported that structured discussions, role-playing, and interpersonal respect activities were effective in decreasing prejudice by enhancing adolescents' empathy and understanding of older persons' societal contributions. Finally, Chen et al. (2021) demonstrated that employing counter-stereotypical scenarios combined with evaluative conditioning techniques (e.g., consistently pairing older persons with positive attributes) led to reductions in implicit age biases among adolescents. This indicates that repeatedly linking positive ideas with older persons can help young people develop less ageist attitudes toward older persons.

## The present study

The present study builds on a prior study by Suberry et al. (2025), who implemented a pre–post-test design—without a control group or follow-up measures—to examine the impact of an educational intervention on ageism among tweens aged 11–15. The intervention, a 90-min creative advocacy and active learning, was delivered to 318 sixth- to ninth-grade students across 25 classrooms (for intervention’s description, see online supplementary material, Appendix A1). Findings indicated that participants exhibited improved attitudes toward older persons, yielding a medium effect size. The intervention was more effective among girls. Analysis of 274 student-created memes revealed that 64% addressed ageism toward older persons, 19% promoted an age-inclusive society, and 17% focused on ageism directed at children (see also Okun et al., 2025).

Two recent meta-analyses have examined the effectiveness of educational interventions to reduce ageism, revealing important differences based on research design (Apriceno & Levy, 2023; Burnes et al., 2019). While Burnes et al. (2019) found no significant difference between quasi-experimental studies and randomized controlled trials (RCTs), Apriceno and Levy (2023) reported that quasi-experimental studies demonstrated significantly stronger effects, with RCTs failing to produce significant results. This raises concerns about the robustness or generalizability of the findings from quasi-experimental studies, which are less controlled than RCTs, while highlighting the potential of longer educational efforts.

Expanding on this prior work, the current study sought to evaluate the efficacy of a similar ageism-reduction intervention while addressing several methodological shortcomings of the original research (Suberry et al., 2025). First, the research design, hypotheses, and analysis plan were preregistered to ensure transparency and reduce bias (https://osf.io/7p3hj). Second, we implemented an RCT design, with data collected at three time points: a week before the intervention, immediately after, and at a 2.5-month delayed follow-up. Participants were allocated to either an intervention or control group. Another advantage of the present study concerns the demographics of the participants, who were both Israeli Jews and Arabs, rather than solely Israeli Jews, as was the case in the pilot phase.

The rationale for targeting both Israeli Jews and Arabs stems from the current demographic characteristics of Israel. As of the end of 2024, Israel’s population consisted of approximately 76.9% Jews and 21.0% Arabs (Israel Central Bureau of Statistics, 2024); 83.3% of the Israeli Arabs are Muslims, 9% Druze, and 7.7% Christians. While preserving their language and cultural identity, Israeli Arabs have increasingly integrated key elements of the dominant Israeli culture to better participate in its social, economic, and political spheres (Smooha, 2019). Most have become bicultural, blending traditional customs with modern values, such as embracing Western ideals like financial independence and competitiveness. These broader modernization and urbanization processes also involve rising levels of education, increased participation of women in the workforce, delayed marriage, and a gradual shift away from traditional ways of life (Veronese et al., 2011).

In traditional Arab society, younger generations were expected to respect and care for older persons, but these obligations have weakened within modern industrial settings. Supporting this view, Litwin and Zoabi (2003) found that older Arab Israeli persons who suffered abuse were significantly more socially isolated than nonabused older persons—particularly in urban environments. A national survey of Israelis aged 50 and older found that Israeli Arabs report higher levels of age discrimination compared to both Israeli Jews and immigrants from the former Soviet Union (Ayalon & Cohn-Schwartz, 2022). Moreover, in a related component of the current research project, and in contrast to findings among Israeli Jewish middle school students (Okun et al., 2025; Suberry et al., 2025), most Israeli Arab students—despite receiving explicit instructions to combat ageism through meme creation—produced memes that depicted old age and aging in a negatively stereotypical manner (Ayalon et al., 2025b).

Nevertheless, other studies reflect the persistence of traditional values in Arab society. For example, Bergman et al. (2013) found that Israeli Arabs reported more favorable cultural attitudes toward older persons, spent more time with them, and viewed them as contributing more to society compared to their Jewish peers. Similarly, Manor (2020) identified strong expectations of filial piety and family caregiving obligations, suggesting that despite ongoing modernization, respect for older persons remains a deeply embedded norm in the Arab culture.

An important aspect of the present research involves its measurement tools. We used measurements that were adapted for a non-English-speaking country, addressing the limitation that many ageism studies are conducted in Western, English-speaking countries (Ayalon et al., 2019). Moreover, measures were explicitly designed to overcome gaps identified by Ayalon et al. (2019), who highlighted significant shortcomings in existing ageism scales concerning their tendency to focus predominantly on stereotypes, neglecting the dimensions of prejudice and discrimination.

Moreover, although the main purpose of the intervention was to target ageism toward older persons, we also assessed attitudes toward younger persons, as our pilot study indicated that the tweens best related to the concept of ageism via their own personal experiences (Okun et al., 2025). In addition, we assessed attitudes toward age inclusivity to capture the overall effect of the intervention beyond one’s particular age.

## Hypotheses

Compared with the control group, we expected participation in the educational intervention to demonstrate:

**H1.** A shift toward more positive and fewer negative or neutral words when describing older persons.

**H2.** Improved attitudes, emotions, and behaviors toward older persons.

**H3.** Greater agreement with the concept “a world for all ages.”

**H4.** Improved attitudes and emotions toward peers (tweens).

Given cultural differences between Israeli Jews and Israeli Arabs, we examined potential sector differences (e.g., Israeli Jews vs Arabs) in the effects of the intervention compared with the control on all outcome variables. However, we posed no prior hypotheses in the absence of past research.

## Method

### Participants

Data for this study were collected as part of the administration of the educational intervention, “It is for our age.” For additional details about this intervention, refer to the online supplementary material, Appendix A1, as well as Suberry et al. (2025) and Okun et al. (2025). The research was approved by the researchers' University Ethics Committee (no. 82203) and by the Chief Scientist at the Israeli Ministry of Education (no. 13467 from September 20, 2023). All participants and their parents signed an informed consent before being included in the analyses. Those who did not provide informed consent (*N *= 3) were granted the opportunity to participate in the intervention, but their data were not collected. We employed a RCT design at the class level within each school in the Jewish sample. Using a computerized lottery system (https://www.random.org/lists/), the first author assigned entire classes—not individual students—to either the intervention or control condition. This was done because the intervention was implemented at the class level. However, in the Arab sample, randomization was implemented at the grade level due to logistic constraints such as class schedules, teacher assignments, and the physical setup of schools. Each Israeli Arab school consisted of four classes that participated in the study: two classes from the same grade were assigned to the intervention group, while two classes from a different grade were designated as the control group. The intervention was administered between April and June 2024.

A power analysis for detecting an effect size of 0.50 with a power of 0.95 and alpha level of 0.05 yielded a minimum sample size of 45 for a between-subject comparison, and 176 participants for a within-subject comparison. We oversampled to account for possible attrition.

The study recruited a total of 616 middle school students from five schools, consisting of 23 classes with sizes ranging from 21 to 37 students (*M *= 26.87). Participants were drawn from two Jewish schools and three Arab schools: two located in central Israel and three in northern Israel, across Grades 7–9 (see online supplementary material, Appendix A1, Table A). Of the participants, 10 were excluded from the analysis due to the following reasons: 3 did not provide valid consent, 4 submitted incorrect identification numbers, and 3 failed to complete the questionnaires. As a result, the final sample comprised 606 students (*M* age = 13.22, *SD *= 0.94; ages ranged from 12 to 16, with 53.3% girls) who were randomly assigned to either the intervention group (*N *= 314) or the control group (*N *= 292). Within the study, 12 classes participated in the intervention while 11 classes served as the control group. Of the total participants, 265 completed all three measurement points (intervention: 136; control: 169), while 341 did not (intervention: 178; control: 163). Among those who did not complete all measurements, 338 completed the pretest (intervention: 177; control: 161) but did not complete the post-tests, and three participants failed to complete the pretest (intervention: 1; control: 2). Table B (see online supplementary material, Appendix A1) provides a summary of the sample sizes at each measurement point (Time 1 = pretest, Time 2 = posttest, Time 3 = follow-up) by sector (Israeli Jews, Israeli Arabs).

Independent sample *t*-tests were performed to assess age differences between the intervention and control groups. Results revealed a significant difference, (*t*(530) = −9.29, *p* < .001), with the average age of the intervention group (*M *= 13.53, *SD *= 1.05) being higher than that of the control group (*M *= 12.88, *SD* = 0.65). Additionally, a significant difference (*t*(510.85) = −16.48, *p* < .001) indicated that the average age of the Arab group (*M *= 13.68, *SD* = 0.97) was higher than that of the Jewish group (*M *= 12.67, *SD* = 0.51). Consequently, all subsequent analyses were conducted with age as a covariate.

Additionally, χ^2^ test of independence was conducted to assess whether there were differences in gender distribution between the groups (intervention vs control) or across sectors (Israeli Jews vs Israeli Arabs). The analyses showed no significant differences in gender distribution between the intervention and control groups (χ^2^ (1) = 0.185, *p* = .667; see Table 1) or between the sectors (Jews vs Arabs; χ^2^ (1) = 1.24, *p* = .265; see Table 2), indicating comparable gender representation across groups and sectors. A χ^2^ test of independence was conducted to examine the relation between sector and study condition. The association was not statistically significant, χ^2^(1) = 1.78, *p* = .182, indicating that participants were comparably distributed across conditions in both sectors.

**Table 1:** Baseline demographics.

Character	Intervention (*n* = 314)	Control (*n* = 292)	χ**^2^ */t***	*p*
**Age**	13.53 ± 1.05 (12–16)	12.88 ± 0.65 (12–14)	−9.29	>.001^a^
**Gender**				
** Boys**	144 (45.9)	139 (47.6)	0.185	.667^b^
** Girls**	170 (54.1)	153 (52.4)		

*Note.* Data are *n* (%), mean ± *SD* (range).

a Independent-sample *t*-test.

b Chi-square test.

**Table 2:** Demographic characteristics by sector.

Sector	Jews (*n* = 278)	Arabs (*n* = 328)	χ^2^ */t*	*p*
**Age**	12.67 ± 0.51 (12–14)	13.68 ± 0.97 (12–16)	−16.48	>.001^a^
**Gender**				
** Boys**	123 (44.2)	160 (48.8)	1.24	.265^b^
** Girls**	155 (55.8)	168 (51.2)		

*Note.* Data are *n* (%), Mean ± *SD* (range).

a Independent-sample *t*-test.

b Chi-square test.

For additional analyses regarding differences in age, gender, and condition of participants who completed all three measurement time points and those who did not, see online supplementary material Appendix A2.

### Measures

Participants reported their age and gender (either “boy” or “girl”).

#### Descriptions of older persons

We administered a word association task concerning older persons. Following past research (Laney et al., 1999; Lichtenstein et al., 2003), we collected qualitative data based on an open-ended question asking participants to list three words that first come to mind when they think about an older man or woman. Arabic responses were translated by a native Arabic speaker. After a discussion among the research team to ensure conceptual clarity and consistency, responses were categorized. Each response was then coded for emotional valence using a three-level scale: 0 = negative (e.g., scary, crazy), 1 = neutral (e.g., adult, age), and 2 = positive (e.g., respect, wisdom). A total score reflecting the participant’s valence toward older persons was calculated by summing the numeric values at each measurement point, with a higher score indicating more positive valence.

#### Attitudes toward older persons/tweens

The Children’s Attitudes Towards Elderly (CATE) questionnaire (Seefeldt et al., 1977) was translated into Hebrew and Arabic and modified from a five-point Likert-type scale to a six-point Likert-type scale by adding an additional middle option. This modification was made to accommodate developmental variability in middle school students. Participants rated 10 items, each representing a continuum of 2 opposing traits of older persons/tweens (“Older persons/My peers are…”). Responses range from 1, indicating the most negative attitudes (“very sad”), to 6, indicating the most positive attitudes (“very happy”). The seventh point was labeled as “I don’t know,” allowing participants who are unable to decide between a negative or positive association to express their uncertainty. The 10 items were averaged, with a higher score reflecting a more positive attitude toward older persons/tweens.

#### Valence thermometers

Valence thermometers were used to assess emotions toward (1) older persons, (2) grandparents, and (3) tween peers. Participants were asked, “How do you feel toward older persons/grandparents/peers?” on a scale ranging from 0 (*negative emotion*) to 100 (*positive emotion*). This procedure follows previous prejudice-reduction research conducted in Israel (Weiss et al., 2023), where similar thermometers were used to measure intergroup affect toward different minority groups (e.g., immigrants, visually impaired children).

#### Behavioral intention to engage with older persons

Behavioral intention to engage with older persons was assessed by a single item. Participants rated “How interested are you in participating in joint activities with your peers involving older persons?” on a Likert scale ranging from 1 (“not interested”) to 10 (“very interested in participating”). Similar single-item intention measures were used in the Israeli field experiments by Weiss et al. (2023) to assess students' willingness to interact with outgroups.

#### A world for all ages index

Three items assessed participants' attitudes toward the inclusivity of people of all ages. For example, “I can learn many new things from people of all ages” using 6 Likert-scale (0 = “strongly disagree” to 6 = “strongly agree”). The three items were averaged, with a higher score representing greater inclusivity of people of all ages. The structure and tone of these items were inspired by Weiss et al.’s (2023) “diversity index,” which similarly combined multiple Likert-style statements reflecting openness toward outgroups.

The reliability of the scales (Cronbach’s alphas) for the three measurement points ranged from acceptable to very good, as shown in Table C (see online supplementary material, Appendix A2).

### Procedure

Each participant was assigned a unique registration number that was used throughout the study. Participants answered an online web-based questionnaire in Qualtrics using their mobile phones. Participants, regardless of their assignment to intervention or control, completed the same questionnaire a week before the intervention began (pretest), immediately after the intervention (post-test), and on average 10.5 weeks following the intervention (follow-up).

### Data analysis

The statistical analyses were performed using R, Excel, and SPSS 29 (IBM Corporation, 2020). Both parametric and non-parametric statistical methods, including independent sample *t*-test and Chi-square test, were applied to compare the baseline demographic characteristics between conditions and sectors. We also conducted a MANCOVA to compare the intervention and control groups on each outcome at T1, controlling for age.

To test H1, we performed a generalized linear mixed-effects model (GLMM) with a Poisson distribution. The dependent variables were the number of positive and negative words used by participants to describe older persons. To test H2, H3, and H4: whether attitudes, emotions, and behaviors toward older persons and toward peers and the agreement with the concept “a world for all ages” were improved in the intervention arm versus the control arm, repeated measures ANOVAs were conducted with age included as a covariate. We focused on within-subject effects over time and between-subject effects based on the research arm. Post-hoc tests were conducted when applicable, and significant results emerged.

Finally, although we did not have a priori hypotheses, we used an exploratory approach to investigate differences between the two sectors (e.g., Israeli Jews vs Israeli Arabs). We examined a 3-way interaction (Times × Sector × Condition) for each outcome variable.

## Results

To test H1, which proposed that the intervention would increase positive and reduce negative descriptions of older persons, we fitted GLMMs with Poisson distributions (see Tables 3 and 4). The models included Time (pre, post, follow-up), Condition (intervention vs control), and their interaction, with age as a covariate.

**Table 3:** GLMM with a Poisson distribution: Results for positive word count.

Predictor	Estimate (β)	*SE*	95% CI		*p*
			*LL*	*UL*	
**Intercept**	−2.33	0.84	−3.97	−0.69	.005
**Time₂**	−0.03	0.13	−0.28	0.22	.838
**Time₃**	−0.13	0.14	−0.39	0.13	.355
**Condition (Intervention)**	0.17	0.14	−0.11	0.45	.231
**Sector (Arabs)**	−0.32	0.15	−0.61	−0.03	.034
**Age**	0.21	0.07	0.07	0.35	<.001
**Time₂ × Condition**	0.00	0.15	−0.29	0.29	.995
**Time₃ × Condition**	−0.16	0.16	−0.47	0.15	.325
**Time₂ × Sector**	−0.06	0.15	−0.35	0.23	.716
**Time₃ × Sector**	−0.05	0.16	−0.36	0.26	.778
**Condition × Sector**	−0.57	0.18	−0.93	−0.21	<.001
**Time₂ × Condition × Sector**	0.15	0.31	−0.46	0.76	.630
**Time₃ × Condition × Sector**	0.10	0.32	−0.53	0.73	.757

*Note*. CI = confidence interval; LL = lower limit; UL = upper limit. Predictors include Time (pre, post, follow-up), Condition (intervention vs control), Sector (Jewish vs Arab), and their interactions.

**Table 4:** GLMM with a Poisson distribution: Results for negative word count.

Predictor	Estimate (β)	*SE*	95% CI		*P*
			*LL*	*UL*	
**Intercept**	1.57	0.84	−0.08	3.22	.061
**Time₂**	0.21	0.17	−0.13	0.55	.220
**Time₃**	0.36	0.16	0.05	0.67	.027
**Condition (Intervention)**	−0.32	0.18	−0.68	0.04	.066
**Sector (Arabs)**	0.32	0.17	−0.01	0.65	.058
**Age**	−0.15	0.07	−0.29	−0.02	.025
**Time₂ × Condition**	0.00	0.18	−0.35	0.35	.996
**Time₃ × Condition**	0.06	0.17	−0.28	0.40	.750
**Time₂ × Sector**	−0.15	0.18	−0.51	0.21	.422
**Time₃ × Sector**	−0.40	0.18	−0.75	−0.05	.028
**Condition × Sector**	0.66	0.19	0.28	1.04	<.001
**Time₂ × Condition × Sector**	−0.59	0.37	−1.32	0.14	.114
**Time₃ × Condition × Sector**	−0.53	0.36	−1.24	0.18	.145

*Note*. CI = confidence interval; GLMM = generalized linear mixed-effects model ; LL = lower limit; UL = upper limit. Predictors include Time (pre, post, follow-up), Condition (intervention vs control), Sector (Jewish vs Arab), and their interactions.

For positive word counts, the Condition × Time interaction was not statistically significant (Time_2_ × Condition: β = 0.00, *SE *= 0.15, *p* = .995; Time_3_ × Condition: β = –0.16, *SE *= 0.16, *p* = .325), indicating that the intervention did not lead to a sustained increase in positive word use over time. There was a significant main effect of age, β = 0.21, *SE *= 0.07, *p* <.001, indicating that older participants were more likely to describe older persons using positive language. A comparable model was tested for negative word counts. Again, the Condition × Time interaction was not significant (Time_2_ × Condition: β = 0.00, *SE* = 0.18, *p* = .996; Time_3_ × Condition: β = 0.06, *SE* = 0.17, *p* = .750), confirming that the intervention did not produce a differential reduction in negative descriptions over time. There was a significant main effect of age, β = −0.15, *SE *= 0.07, *p* = .025, indicating that compared with younger participants, older participants were less likely to describe older persons using negative language.

A MANCOVA was conducted to compare the intervention and control groups on multiple dependent variables at baseline, while controlling for age. The analysis revealed a significant effect of group condition on willingness to participate in intergenerational meetings (*F*(1, 570) = 5.56, *p* = .018, η^2^ = 0.010). Participants in the control group (*M *= 5.87, *SD *= 3.39) reported a significantly higher willingness to participate compared to those in the treatment group (*M *= 5.58, *SD *= 3.24). No other significant differences between the groups were found across the remaining outcome variables.

We further tested H2, whether attitudes toward older persons, emotions regarding both older persons and grandparents, and behaviors toward older persons, improved over time, by comparing the intervention and control groups (see Table 5 for numeric values and Figure A in the online supplementary material Appendix for trends and patterns in data). First, with respect to quantitatively measured attitudes toward older persons (e.g., CATE questionnaire), the results showed no significant effect across time, *F*(2, 240) = 1.15, *p* = .316, *n*^2^  *=* 0.005. Moreover, the results of the between-subject analysis revealed a non-significant interaction between Time and Condition, *F*(2, 240) = 2.12, *p* = .146, *n*^2^  *=* 0.009, indicating that the pattern of change over time was similar for both the intervention and control groups. Regarding emotions toward older persons, the results indicated a significant effect of time, *F*(2, 247) = 4.41, *p* = .014, *n*^2^  *=* 0.018. Post-hoc analyses indicated an initial increase in scores (i.e., more positive emotions) from pretest (*M = *78.33) to posttest (*M = *80.31), followed by a decrease at follow-up (*M *= 79.66). However, the between-subject analysis revealed a non-significant effect, *F*(1, 247) = 0.737, *p* = .391, *n*^2^  *=* 0.003, indicating no differences over time. Regarding emotions toward grandparents, the results indicated no significant effect over time, *F*(2, 240) = 3.02, *p* = .054, *n*^2^ *=* 0.012. Moreover, the results of the between-subject analysis revealed a non-significant interaction between Time and Condition, *F*(2, 240) = 0.003, *p* = .954, *n*^2^  *=* 0.000, indicating no differences in change over time. Behavioral intentions toward older persons, measured by intergenerational contact interest, did not differ over time, *F*(2, 243) = 0.339, *p* = .706, *n*^2^  *=* 0.001, nor between intervention and control groups, *F*(2, 243) = 3.50, *p* = .063, *n*^2^  *=* 0.014. Overall, results failed to support H2 that the intervention group would report improved attitudes, emotions, and behaviors toward older persons.

**Table 5:** Outcome variables across measurement points and research arm.

Outcomes	Time 1		Time 2		Time 3		Main effect over time	Between subjects (intervention vs control)
	Intervention, *n* = 308	Control, *n* = 280	Intervention, *n* = 189	Control, *n* = 206	Intervention, *n* = 189	Control, *n* = 167	Partial, *n* ^2^	Partial, *n* ^2^
**Stereotypes toward older persons**	4.18 ± 0.66 (2.33–6)	4.12 ± 0.73 (1–6)	4.24 ± 0.73 (1–6)	4.09 ± 0.93 (1–6)	4.15 ± 0.81 (1–6)	4.05 ± 0.88 (1–6)	.005	.009
**Emotions toward older persons**	77 ± 21.1 (0–100)	78 ± 24.2 (0–100)	79 ± 21.1 (0–100)	79 ± 23.2 (0–100)	76 ± 24.1 (0–100)	81 ± 22.0 (0–100)	.018**	.003
**Emotions toward grandparents**	91 ± 18.9 (0–100)	9 ± 21.3 (0–100)	89 ± 18.6 (10–100)	91 ± 19.1 (7–100)	88 ± 22.9 (0–100)	92 ± 18.8 (0–100)	.012	.000
**Intergenerational contact interest**	5.54 ± 3.27 (0–10)	5.83 ± 3.42 (0–10)	5.95 ± 3.01 (0–10)	5.94 ± 3.36 (0–10)	5.09 ± 3.32 (0–10)	5.87 ± 3.25 (0–10)	.001	.014
**Inclusive world for all ages**	4.08 ± 0.93 (1–6)	4.09 ± 0.90 (1–6)	4.14 ± 0.93 (1–6)	3.99 ± 1.06 (1.67–6)	4.05 ± 0.97 (1–6)	4.07 ± 1.05 (1–6)	.000	.002
**Stereotypes toward tweens**	4.44 ± 0.86 (1–6)	4.48 ± 0.93 (1–6)	4.42 ± 0.92 (1–6)	4.45 ± 0.97 (1–6)	4.31 ± 0.97 (1–6)	4.39 ± 1.02 (1–6)	.001	.001
**Emotions toward tweens**	62 ± 28.4 (0–100)	62 ± 28.8 (0–100)	63 ± 27.8 (0–100)	64 ± 27.6 (0–100)	61 ± 27.9 (0–100)	64 ± 28.1 (0–100)	.013**	.000

*Note.* Higher scores indicate more positive stereotypes and emotional valance toward older persons, grandparents, and tweens, greater interest in intergenerational activities, and more favorable attitudes toward age inclusivity. Mean ± *SD* (range).

** *p* < .01.

H3 was not supported by the data. The agreement with the concept of “A world for all ages” showed no significant differences over time, *F*(2, 244) = 0.02, *p* = .981, *n*^2^ = 0.000, nor between the intervention and control groups, *F*(1, 244) = 0.37, *p* = .543, *n*^2^ = 0.002. The Time × Condition interaction was also not statistically significant, *F*(2, 244) = 2.57, *p* = .079, *n*^2^ = 0.010, indicating that the intervention did not significantly influence changes in age inclusivity attitudes over time ­compared to the control.

The data did not support hypothesis H4 regarding attitudes toward tweens. No differences were observed over time in stereotypes toward tweens, *F*(2, 237) = 0.25, *p* = .758, *n*^2^ = 0.001. The Time × Condition interaction was also not statistically significant, *F*(2, 237) = 0.27, *p* = .741, *n*^2^ = 0.001. A small effect size regarding changes in emotions toward tweens over time was noted, *F*(2, 247) = 3.32, *p* = .039, *n*^2^ = 0.013. However, follow-up comparisons revealed no significant differences between any of the time points (*p* > .99), and the mean scores remained relatively stable (Time 1: *M *= 65.76; Time 2: *M *= 64.78; Time 3: *M *= 65.05). Thus, while the omnibus test reached significance, there is no clear evidence of meaningful change in participants' emotional responses toward tweens over time. Moreover, the Time × Condition interaction was also not statistically significant, *F*(2, 247) = 0.096, *p* = .900, *n*^2^ = 0.000, indicating no significant intervention-related change in emotions toward tweens over time. Overall, no improvements in attitudes and emotions toward tweens were observed in the intervention group versus control over time, leading to rejection of H4.

### Intervention effects across sectors

An exploratory analysis utilizing GLMMs with a Poisson distribution model tested the number of positive and negative words used to describe older persons across sectors. The models tested three-way interactions between Time, Condition, and Sector, with age included as a covariate. For both the number of negative (*b* = –0.25, *SE *= 0.18, *p* = .162) and positive words (*b *= 0.045, *SE *= 0.159, *p* = .776) used to describe older persons, the three-way interaction effects were non-significant. This indicates that the pattern of change over time in word usage did not significantly differ across sectors or between research arms.

An exploratory analysis assessed the intervention’s effectiveness across the two sectors (Jews and Arabs across time and research arm) by conducting repeated measures ANOVAs. All three-way interactions (Time × Condition × Sector) were non-significant, indicating that patterns of change over time did not differ meaningfully between sectors or intervention conditions (see Table D in the online supplementary material, Appendix).

## Discussion

The present study responds to long-standing calls for increased methodological rigor in ageism reduction research. Unlike pre–post designs, which are more vulnerable to threats such as maturation, testing effects, or regression to the mean, RCTs provide stronger internal validity by controlling for confounding variables and establishing clearer causal inferences. Paluck and Green (2009) reviewed the literature and found that many interventions had not been thoroughly tested using RCTs. In line with this, ageism scholars emphasize that “research on ageism interventions should, where possible, use randomized controlled experiments” (Nelson, 2019, p. 1066). Therefore, we conducted an RCT, relying on a three-point measurement schedule to test the efficacy of an intervention, which was successfully tested in a pre–post pilot test involving 318 middle school students (Suberry et al., 2025).

Moreover, in addition to addressing the need for methodological rigor using an RCT, the present study sought to expand the scope of measurement by examining not only cognitive components of ageism (i.e., stereotypes) but also affective (prejudice) and behavioral (discrimination) dimensions. A key strength of the present study is its use of culturally adapted measures in both Hebrew and Arabic that captured all three dimensions of ageism—stereotypes, prejudice, and discrimination. These tools demonstrated good to excellent reliability, addressing known limitations of previous scales and broadening their relevance beyond English-speaking contexts (Ayalon et al., 2019). Additionally, the study employed methodological triangulation by using both a quantitative questionnaire (Seefeldt et al., 1977) and a qualitative open-ended word association task to assess attitudes toward older persons, which was later transformed into quantitative data for the purpose of analysis. This dual-measure approach strengthened construct validity.

Furthermore, the study aimed to assess the durability of intervention effects by evaluating outcomes across multiple time points, thereby addressing the critical question of whether short educational programs produce lasting change beyond the immediate aftermath of the intervention. Importantly, the intervention was implemented among both Jewish and Arab middle school students in Israel, allowing for a cross-cultural examination of its effectiveness. Given the sociocultural differences between these two populations—and the potential for variations in perceptions of older persons (Ayalon & Cohn-Schwartz, 2022)—this approach contributes to a more inclusive understanding of how ageism develops and how it may be addressed across diverse societal groups.

Despite its strengths and the fact that the intervention was successfully piloted, the present study did not produce significant changes in age-related attitudes, emotions, or behavioral intentions. There were no significant differences observed between the intervention and control groups, nor were there meaningful changes over time. These null findings prompt critical reflection on the intervention content, as well as contextual and methodological factors that may impair its efficacy.

Several key differences between the current study and the earlier pilot study (Suberry et al., 2025) may help explain the divergence in findings. The pilot employed a pre–post design, with participants completing the questionnaire immediately before and directly after the intervention. In contrast, the present RCT administered preintervention measures approximately 1 week prior to the intervention, while the postintervention assessment was conducted immediately following the session. Although the structure of the intervention remained largely consistent across studies, the current version placed additional information on the mutual benefits of intergenerational connections for both young and older individuals. We intended to promote positive stereotypes of older persons as knowledgeable and experienced. Although theoretically aligned with prior successful interventions emphasizing the benefits of challenging misconceptions about aging (e.g., Chen et al., 2021), this addition did not yield significant attitude changes in the present study.

Additionally, the measurement tools used in the two studies differed. The present study incorporated broader, multicomponent assessments—feeling thermometers toward older persons, grandparents, and peers, plus a behavioral-intention item—whereas the pilot relied primarily on attitudinal items. This broader coverage may have prompted deeper reflection and reduced simple acquiescence, though the concise format (including the use of a single-item indicator) may have limited sensitivity. Notably, as Apriceno and Levy (2023) observed, quasi-experimental studies often report stronger effects than RCTs, likely due to reduced internal validity in less controlled designs—offering further context for the differing outcomes observed across the two phases.

Another important factor that may have limited the intervention’s effectiveness is the context in which the study was conducted—during wartime. The Israel–Gaza war began on October 7, 2023, and escalated into a regional conflict involving multiple fronts. Some of the missile attacks directly targeted communities participating in this study, creating a climate of persistent threat and anxiety. A recent nationwide survey conducted before and after the onset of the war, among both Jewish and Arab populations, found a significant increase in symptoms of post-traumatic stress disorder (PTSD) , depression, and anxiety—particularly among younger individuals—highlighting the war’s psychological toll on youth (Levi-Belz et al., 2024). It is plausible that the distressing wartime environment interfered with students' ability to fully engage with the intervention’s messages about ageism, thereby contributing to the null results observed.

A key limitation and possible explanation for the null findings is the considerable attrition, as many participants did not complete all three measurement waves and were excluded from the final analysis. To assess potential attrition bias, we compared participants who completed all waves with those who dropped out (see online supplementary material Appendix A2). No significant differences were found in age, sector, or research arm, though girls were more likely than boys to complete all surveys. While attrition was not condition- or sector-specific, the high dropout rate and gender imbalance limit confidence in the findings and may partly explain the null results.

Educational interventions have shown efficacy in targeting university students, particularly those enrolled in aging-related courses (Chonody, 2015). Such findings may reflect a selection bias, as these students are often predisposed to hold more positive views of older persons. By contrast, the middle school students who participated in this study were not self-selected and likely approached the intervention without prior positive bias. Moreover, recent reviews highlighted that structured educational content becomes more effective when combined with opportunities for direct or indirect interaction and reflective processes (Apriceno & Levy, 2023; Bétrisey et al., 2024; Burnes et al., 2019). Therefore, future ageism-reduction programs targeting adolescents may benefit from incorporating both explicit educational elements and authentic intergenerational experiences. Nonetheless, our findings thus align with broader concerns regarding the limited impact of brief interventions and suggest a need for more comprehensive and sustained approaches (Apriceno & Levy, 2023).

Given cultural differences between Israeli Jews and Arabs, we explored sector differences across conditions and timepoints, without prior hypotheses due to limited existing research. Our findings indicate that the intervention was equally ineffective among both Jewish and Arab participants. There might be a greater need for interventions in the Arab sector in line with research showing higher levels of ageism among Israeli Arabs compared to Jews (Ayalon & Cohn-Schwartz, 2022). Moreover, Ayalon et al. (2025a,b) showed that after participating in an ageism-reduction intervention, most Arab students produced content reinforcing negative stereotypes about aging. These depictions of older persons as cognitively and physically incapable, associated with death and decay, and even as targets of abuse or ridicule, highlight persistent and severe ageist perceptions among participants.

### Recommendations and future directions

Despite yielding null results, the present study offers meaningful contributions to the science of ageism intervention. Based on available evidence, it is the first RCT to evaluate an educational anti-ageism intervention in a multicultural context among both Israeli Jewish and Arab middle school students. A key strength lies in its use of measures that targeted all three dimensions of ageism—stereotypes, prejudice, and discrimination—in both Hebrew and Arabic speakers. Importantly, it underscores the challenge of eliciting attitudinal, emotional, and behavioral changes among tweens and the potential limitations of brief, education-only interventions. As noted in recent methodological literature (Fanelli, 2012; Tian et al., 2024), null results are essential for reducing publication bias, refining theoretical assumptions, and guiding future research design. By transparently reporting our findings, we help clarify what does *not* work under specific conditions and highlight methodological challenges, thereby offering lessons for researchers developing more effective, contextually grounded interventions against ageism.

Intervention efficacy may be enhanced by transitioning from brief, education-only sessions to a multisession, spaced curriculum with periodic booster activities, which address intergenerational contact. Drawing on intergenerational education literature, it is beneficial to combine educational contents with real-life experiential activities, which involve intergenerational contact (Guardabassi, 2025) around equal status, shared goals, and cooperative tasks—such as technology-tutoring sessions where tweens mentor older persons in digital skills while older persons reciprocate by sharing experiential knowledge and life narratives (Gamliel & Gabay, 2014). Incorporating arts-based, experiential components (music, movement, storytelling) that resonate with tweens may also be beneficial in reducing ageism (Kim & Liao, 2025; Suberry & Bodner, 2024). Likewise, capitalizing on co-design processes with students and teachers to enhance the relevance and feasibility of the interventions and embed the content within existing curricula (e.g., civics, language) is desirable. Finally, it is potentially desirable to target different stakeholders, including students, classes, schools, and families, by pairing students' lessons with teachers' professional development activities, family conversation prompts, and school-climate activities.

## Supplementary Material

gnaf274_Supplementary_Data

## Data Availability

The materials and data from this study are available as supplemental material at https://osf.io/un5f9/files. The study reported in the article was preregistered at https://osf.io/7p3hj.
