# Supplementary material for: Reducing ageism among Israeli Jew and Arab middle school students: a randomized controlled trial
Source: Gerontologist. 2025 Nov 23;66(4):gnaf274. doi: 10.1093/geront/gnaf274 (PMC13017870; doi:10.1093/geront/gnaf274)
Supplement: gnaf274_Supplementary_Data [file gnaf274_supplementary_data.docx]

**Supplemental File and Online Appendices:**

**Reducing ageism among Israeli Jew and Arab middle school students: A randomized controlled trial**

Assaf Suberry, Sarit Okun, and Liat Ayalon

Louis and Gabi Weisfeld School of Social Work, Bar-Ilan University, Ramat Gan, Israel.

**Corresponding Author:**

Assaf Suberry, Louis and Gabi Weisfeld School of Social Work, Bar-Ilan University, Israel.

E-mail: [assaf.su@gmail.com](mailto:assaf.su@gmail.com)

**A1. Intervention description**

The educational workshop, “It Is for Our Age,” is a 90-minute interactive session designed to combat ageism among tweens. It is described in detail elsewhere (Okun et al., 2025; Suberry et al., 2025). The intervention was guided by trained university students and was delivered in Hebrew by Jewish university students and in Arabic by Arab university students, all with social sciences backgrounds. Prior training and ongoing supervision were provided to group moderators. To ensure cultural relevance of the intervention and measures to the Arab sector (as the pilot stage took place in the Jewish sector), the intervention and measures were translated and reviewed by a professional translator and by Israeli Arab students.

The intervention is structured around a 24-slide PowerPoint presentation that incorporates pedagogical strategies to stimulate discussion and reflection on aging and ageism. It also includes exposure to information about ageism. It begins with a slide presenting both positive and negative stereotypes commonly associated with older persons. Another early slide displays eight photographs illustrating the heterogeneity of aging—for example, a grandfather engaged in play with his young grandchildren, or an older woman receiving care in a hospital setting. An interactive component follows, where participants respond to 12 statements using stickers to indicate agreement or disagreement (items reported elsewhere; Ayalon, Okun, et al., 2025). Additional slides define ageism, explain how it manifests across various societal domains (such as media, healthcare, and employment), highlighting its harmful consequences for the health and well-being of older persons. Four slides highlight the mutual benefits of intergenerational relationships, both within and beyond the family, presenting them as a “win–win situation.” This segment also includes an 8-minute video featuring older persons reflecting on their experiences of aging. The final segment engages students in an applied activity: creating memes through an online meme generator, designed to challenge ageist narratives and promote age-inclusive messages (Okun et al., 2025).

**Table A**

*Intervention and Control Participants by School, Grade and Sector*

| Class Number | School | Grade | Sector | Intervention (n) | Control (n) |
| --- | --- | --- | --- | --- | --- |
| 1 | A | 7 | Jewish | 31 |  |
| 2 | A | 7 | Jewish | 21 |  |
| 3 | A | 7 | Jewish |  | 30 |
| 4 | A | 7 | Jewish | 24 |  |
| 5 | A | 7 | Jewish |  | 29 |
| 6 | A | 7 | Jewish |  | 14 |
| 7 | B | 7 | Jewish | 28 |  |
| 8 | B | 7 | Jewish | 29 |  |
| 9 | B | 7 | Jewish |  | 28 |
| 10 | B | 7 | Jewish | 21 |  |
| 11 | B | 7 | Jewish |  | 23 |
| 12 | C | 8 | Arab | 27 |  |
| 13 | C | 8 | Arab | 22 |  |
| 14 | C | 7 | Arab |  | 28 |
| 15 | C | 7 | Arab |  | 26 |
| 16 | D | 9 | Arab | 37 |  |
| 17 | D | 9 | Arab | 27 |  |
| 18 | D | 8 | Arab |  | 33 |
| 19 | D | 8 | Arab |  | 29 |
| 20 | E | 9 | Arab | 26 |  |
| 21 | E | 9 | Arab | 21 |  |
| 22 | E | 7 | Arab |  | 25 |
| 23 | E | 7 | Arab |  | 27 |

**Table B**

*Sample Size in Each of the Three Measurement Points by Sector: Israeli Jews and Israeli Arabs*

| Time 3 | Tine 2 | Time 1 |  | Sector |
| --- | --- | --- | --- | --- |
| 108 | 105 | 124 | Control | Israeli Jews |
| 134 | 107 | 154 | Intervention |  |
| 242 | 212 | 278 | Total |  |
| 125 | 149 | 168 | Control | Israeli Arabs |
| 135 | 128 | 601 | Intervention |  |
| 260 | 277 | 328 | Total |  |
| 502 | 489 | 606 | Overall Sample |  |

**A2. Participants characteristics**

To examine the differences between individuals who completed three measurement-time points (Group A) and those who did not (Group B – dropped after pretest; Group C – did not fill pretest), three analyses were conducted: One-Way ANOVA (for age) and c (for gender and research arm). The results of a One-Way ANOVA indicated no significant difference between the survey completion groups (F(2, 603) = .315, *p* =.730). A χ^2^ test of independence revealed a significant difference in gender distribution among the survey completion groups (*χ*^2^ (2) = 8.23 , *p* = .016). These results indicate that fewer boys (n = 110) than girls (n = 155) completed all surveys, while a higher numbers of boys dropped out (n = 173) compared to girls (n = 165). Finally, a χ^2^ test of independence showed no significant difference in research arm (intervention vs control) among the survey completion rates (*χ*^2^ (2) = 4.78, *p* = .788), which were similar across intervention and control arms.

To examine the differences of survey completion between Jewish and Arabs participants, a chi-square test of independence was conducted. The test revealed no significant association between Sector (Jewish vs. Arab) and survey completion group (completed all waves, dropped after pretest, or did not fill pretest), χ²(2) = 0.76, *p* = .684. This suggests that the survey dropout patterns did not differ meaningfully between sectors.

**Table C**

*Cronbach’s Alpha for Stereotypes toward Older Persons/Tween and Inclusivity Questionnaires at Three Time Points*

| Measurement | Time 1 | Time 2 | Time 3 |
| --- | --- | --- | --- |
| Stereotypes toward older persons | .789 | .879 | .864 |
| Stereotypes toward tweens | .855 | .893 | .874 |
| Inclusive world for all ages | .661 | .762 | .765 |

*Note.* Cronbach's Alpha reliability ratings: .60 ≤ α < .70 (Acceptable), .70 ≤ α < .80 (Good), .80 ≤ α < .90 (Very Good).

**Table D**

*Outcome Variables Across Measurement Points by Research Arm and Sector (Three-Way Interaction: Time × Condition × Sector)*

| Outcomes | df | F | *p* | Partial *n^2^* |
| --- | --- | --- | --- | --- |
| Stereotypes toward older persons | 2 | 1.24 | .291 | .005 |
| Emotions toward older persons | 2 | 2.31 | .100 | .009 |
| Emotions toward grandparents | 2 | .77 | .460 | .003 |
| Intergenerational contact interest | 2 | 2.67 | .070 | .011 |
| Inclusive world for all ages | 2 | .76 | .467 | .003 |
| Stereotypes toward tweens | 2 | 1.77 | .172 | .007 |
| Emotions toward tweens | 2 | 1.55 | .213 | .006 |

**Figure A**

*Visual Representation of Repeated Measures ANOVA Results for the Entire Sample*

| **A** | **B** |
| --- | --- |
| **C** | **D** |
| **E** | **F** |
| **G** | 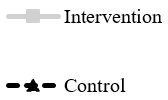 |

*Note.* Outcome means across three measurements of intervention and control groups. Higher scores indicate more positive stereotypes and emotional valance toward older persons, grandparents and tweens, greater interest in intergenerational activities, and favorable attitudes toward age inclusively. All analyses were conducted with age as a covariate.
